# Hyperspectral Imaging (HSI) in anatomic left liver resection

**DOI:** 10.1016/j.ijscr.2019.08.025

**Published:** 2019-08-31

**Authors:** Robert Sucher, Alvanos Athanasios, Hannes Köhler, Tristan Wagner, Maximilian Brunotte, Andri Lederer, Ines Gockel, Daniel Seehofer

**Affiliations:** a Department of Visceral-, Transplant-, Thoracic- and Vascular Surgery, University Clinic Leipzig, Germany; b Innovation Center Computer Assisted Surgery, University of Leipzig, Germany

**Keywords:** Liver resection, Intraoperative visualization, Hyperspectral imaging, Surgical guidance

## Abstract

**Introduction:**

Anatomic liver resection is based on the description of functional segments, which rely on the organs arterial and portal venous blood supply. Vascular inflow control of the left liver is performed by occlusion of the left hepatic artery (LHA) and left portal vein (LPV). Depending on the quality of the parenchyma a sharp demarcation line (Cantlie Line) between segments IV and V/VIII can hence be detected.

**Material and Methods:**

TIVITA® is a novel contact free tool which facilitates non-invasive hyperspectral imaging (HSI) and near infrared spectroscopy (NIRS) for the assessment of tissue- oxygenation (StO_2_) and perfusion (NIR Perfusion). We hypothesized that this imaging modality might be practicable to identify the future resection plane after left vascular inflow occlusion in anatomic liver resection.

**Presentation of Case:**

TIVITA ® is a viable tool for the identification of segments with reduced StO_2_ (inflow occlusion: 0.23 ± 0.03 vs normal: 0.50 ± 0.06) and NIR Perfusion (inflow occlusion: 0.02 ± 0.04 vs normal: 0.47 ± 0.06) and allows for a visual differentiation of well oxygenated, perfused (green) and low oxygenated, poorly perfused (blue) liver tissue in a patient undergoing left hemihepatectomy for hepatocellular carcinoma.

**Conclusion:**

Hyperspectral Imaging is an emerging optical technique with the potential to identify exact resection planes for anatomic liver resection based on the optically determined perfusion and oxygenation status of liver segments lined up for resection. This novel Hyperspectral Demarcation Technique (HSI DT) is non- contact, non-ionizing and non-invasive.

## Highlights

• Hyperspectral Imaging (HSI) is non-contact, non-ionizing and non-invasive.
• HSI detects tissue pathology based on spectral characteristics of different tissues.
• HSI is well suited for counter perfusion staining in anatomic liver resection.
• HSI can be used for future image guided liver surgery.

## 1 Introduction

The work presented here has been reported in line with the SCARE criteria and PROCESS criteria [1].

Novel intraoperative imaging techniques have the potential to transform the way surgery is performed today. Based on the spectral characteristics of different tissues, spectroscopy and hyperspectral imaging (HSI) is capable to offer non-contact, non-invasive and non-ionizing quantitative diagnostic information about tissue pathology, morphology and chemical composition [2].

One of the leading systems for medical hyperspectral imaging is TIVITA® (Diaspective Vision, Am Salzhaff, Germany). The technology behind TIVITA® is based on the principle of imaging spectroscopy. In brief, the camera system incorporates a high number of spectrally differentiated channels, which detect the light which is reflected from the examined object. The object's chemical composition can hence be evaluated on the basis of the recorded wavelength. The camera system is not only capable to detect wavelength within the spectrum of visible light, but also catches wavelength within the near infrared spectrum (NIR), which is invisible to the human eye. Images provided for the surgeon accordingly comprise normal color pictures of the object as well as further processed illustrations which have gone through a “chemical color imaging procedure”. This method allows the reproduction of pictures indicating the Oxygen Saturation (StO_2_), Tissue Perfusion (NIR, Near Infrared Perfusion Index), Hemoglobin (OHI, Organ Hemoglobin Index) and Water concentration (TWI, Tissue Water Index). HSI can be applied almost “real time” and the acquisition for the hyperspectral image takes less than 10 s.

Applied intraoperatively HSI may have a key impact on surgical guidance through tissue characterization. Several surgical disciplines, including reconstructive surgery [3], urology [4,5], surgical oncology [6,7] and neurosurgery [8] have used this technology with a proven record of success for intraoperative tumor detection, quantitative measurement of biomarkers and tissue perfusion assessment [9]. To our knowledge, however, it has not been used in the field of hepatobiliary surgery.

Major hepatectomy is defined as the resection of three or more liver segments which are connected to each other [10]. According to the Couinaud nomenclature a left hemihepatectomy includes the resection of liver segments II-IV [11]. The important anatomic landmark between the right and the left lobe of the liver is the middle hepatic vein, which in most instances can be visualized by ultrasound in the midplane of the liver.

Vascular inflow control of the left liver is performed by occlusion of the left hepatic artery (LHA) and left portal vein (LPV). Depending on the quality of the parenchyma a sharp demarcation line (Cantlie Line) between segments IV and V/VIII can hence be detected on the liver surface, since ischemic segments display marked color differences when compared to normally perfused segments which are not affected by inflow occlusion [12]. This technique also referred as “conventional demarcation technique” (CDT) marks only the organ surface and sometimes fails to execute a completely valid demarcation.

## 2 Case report

A 63-year old male patient underwent cholecystectomy for symptomatic cholecystolithiasis in a peripheral hospital. Apart from a child A cirrhotic liver parenchyma, preoperative ultrasound did not detect any suspect intrahepatic lesions. However, intraoperative findings indicated the presence of 3 suspect intrahepatic lesions within the left liver segments 2 and 3 and 4. An effort was made to resect one lesion for further biopsy. Frozen sections were positive for hepatocellular carcinoma (HCC) with R1 resection margins. Subsequently further liver resection attempts were not made, and the operation was terminated after cholechystectomy. The patient was subsequently transferred to our hospital.

**Fig. 1:**
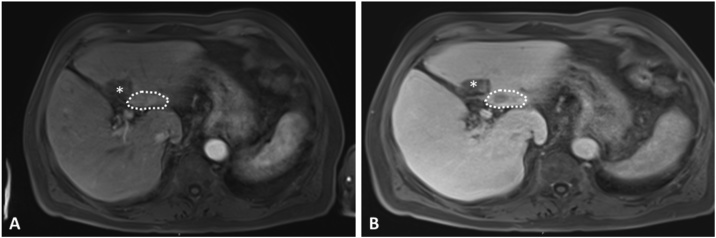
Primovist MRI images indicating lesions within the left liver suspect for HCC (scattered circle) and lesion of previous R1 resection (*).

Primovist contrast enhanced MRI scans (Fig. 1 A, B) indicated the presence HCC suspect lesions within segments 2, 3 and 4. Preoperative liver function assessment by LIMAx [13] revealed no contraindication for the planed left anatomic liver resection. The procedure was performed after informed consent of the patient 6 days post cholecystectomy.

## 3 Surgical procedure

In brief, the patient was positioned supine with both arms extended. Following a team timeout and under sterile conditions a right subcostal incision with an upper midline extension was performed. Exposure was obtained by a Rochard surgical retractor and the abdominal cavity was first explored to rule out undiagnosed extrahepatic disease. Intraoperative ultrasound facilitated visualization of the HCC tumor nodules in segments II,III and IV and the decision was made to proceed with the planed anatomic left liver resection.

**Fig. 2:**
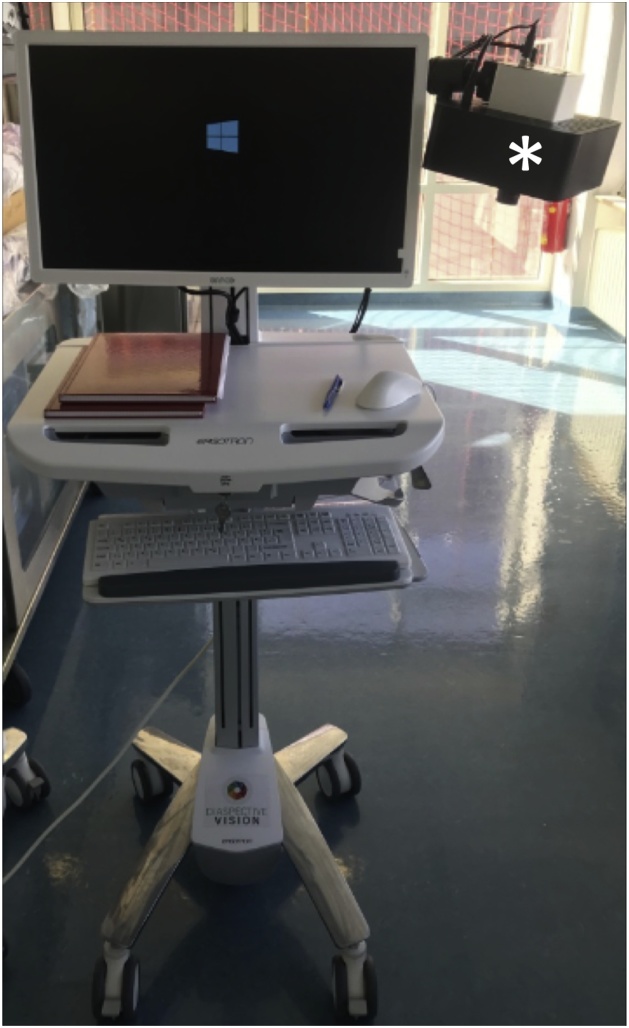
TIVITA® device with light source and HSI camera (*). The chemical color imaging procedure is calculated with the attached computer.

**Fig. 3:**
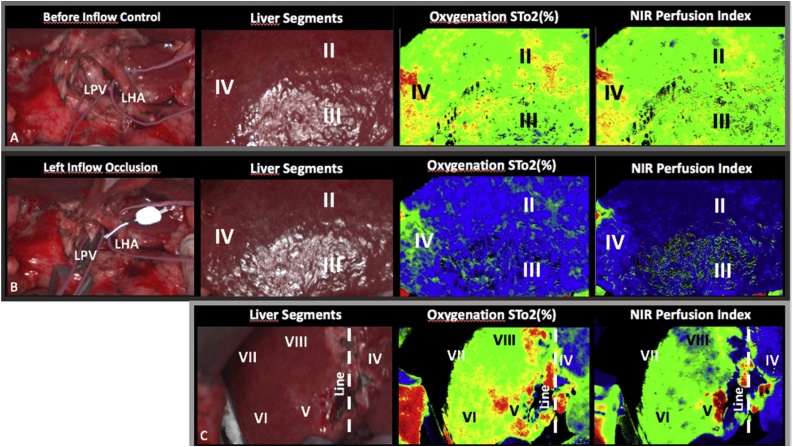
Hyperspectral images of the left liver with color coded oxygen distribution and near infrared perfusion index (NIR) before (A) and after (B) left inflow occlusion.

**Fig. 4:**
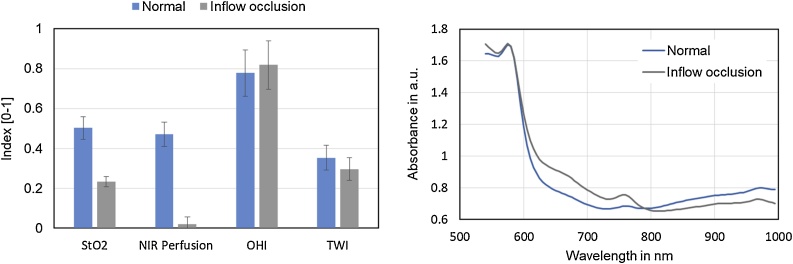
Distribution of tissue parameters before and during inflow occlusion within the liver segments II - IV (left). Mean absorbance spectra of liver segments II and IV with normal perfusion and after inflow occlusion.

In a first step, the round and faciform ligament were dissected up to the anterior surface of the hepatic veins. The gastrohepatic ligament was opened and the caudate lobe exposed. As determined by preoperative MRI a normal Michels type 1 hepatic arterial anatomy was confirmed intraoperatively [14]. Systematic lymphadenectomy of the falciform ligament was performed and frozen sections revealed no malignant lymph node infiltration. Once the left hepatic artery and branch of the left portal vein were dissected free, left vascular inflow occlusion was conducted with Yasargil and Bulldog Clamps. Arterial and portal venous vascular clamping did not result in marked color differences of the liver surface of ischemic segments implying a fail of the conventional demarcation technique. However, before and after inflow occlusion the liver was examined with the TIVITA® hyperspectral camera system (Fig. 2). The camera acquired RGB pictures of the liver before and after left inflow occlusion and imaging software generated pictures illustrating oxygen saturation (StO_2_) and Tissue Perfusion (NIR P, Near Infrared Perfusion index) of the liver specimen lined up for resection. As depicted in Fig. 3 B, left inflow occlusion resulted in significant reduction of tissue oxygenation and perfusion of liver segments II, III, and IV (color coded pictures show a blue liver surface) when compared to normal perfusion (Fig. 3A, color coded pictures show a green liver surface). A larger overview image of the entire liver (Fig. 3C) furthermore displayed a clear difference of oxygenation and perfusion between the left (liver segments II, III, IV) and the right liver (liver segments V-VIII) and traced the future line for parenchymal transection. Fig. 4 shows the distribution of the four calculated tissue parameters before and during inflow occlusion within the liver segments II and IV. The corresponding mean absorbance spectra are illustrated as well.

In a next step the middle hepatic vein, a landmark structure for anatomic liver resection, was visualized by intraoperative ultrasound and the transection line according to the Cantli line line was marked on the liver surface with diathermia. This line perfectly matched with the transection line acquired by HSI imaging. Finally, the parenchymal transection was performed with the Cavitron Ultrasonic Surgical Aspirator (CUSA). Total operative time was 218 min, with an estimated blood loss of less than 200 ml. No Pringle maneuver (total vascular inflow control) was performed during this procedure. Once surgery was completed the patient was transferred to the intensive care unit for one night. The further postoperative course was uneventful, and the patient could be discharged home on post-operative day nine.

## 4 Discussion

Here we describe the application of a Hyperspectral Imaging (HSI) device during anatomic liver resection. Following a failed conventional demarcation technique (CDT), we were able to acquire color coded pictures of normally perfused/oxygenated liver segments and of liver segments which were poorly perfused/oxygenated following left vascular inflow occlusion. The sharp transition zone between ischemic and normally perfused liver segments literally determined our future anatomic transection line and plane.

This “hyperspectral demarcation technique” (HSI DT) is particular valid for anatomic liver surgery since liver anatomy is based on the description of functional segments, which rely on the organs arterial and portal venous blood supply.

In contrast to normal human vision, HSI provides the surgeon with advanced visualization of the liver which is extended into the infrared (IR) and near-infrared (NIR) wavelength regions. In line, this technical approach facilitates in depth understanding of blood oxygen delivery and hypoxia at a microvascular level during liver resection performing vascular inflow control prior parenchymal transection.

Modern liver surgery not only requires expert skills but also an explicit understanding of liver anatomy and real time navigation during parenchymal transection. Resections guided by a demarcation line after portal and arterial inflow clamping of the target territory were established as a fundamental method for anatomical liver resection [15]. Recently, indocyanine green imaging techniques have been described which not only provide demarcation of the liver surface but also facilitate a 3-dimensional parenchymal staining of the target territory [16].

Hyperspectral imaging basically provides same features and does not require introduction of agents. It is an exclusively non-contact, non-ionizing and non-invasive procedure.

On the basis of this study we advocate the use of HSI for open anatomic liver resections where conventional demarcation techniques fail following vascular inflow occlusion. Hyperspectral determination of liver demarcation was applied safely and successfully to anatomic left liver resection and the efficacy of this novel navigation technique was clearly demonstrated.

## Funding

There are no sponsors and there was no special funding for this case report. We acknowledge support from the German Research Foundation (DFG) and Leipzig University within the program of Open Access Publishing.

## Ethical approval

The ethical approval for the publication of this case was exempted by our institution because all of the data were collected from clinical records and imaging systems for routine perioperative planning.

## Consent

Written and informed consent was taken from the patient for publication of this case report and the accompanying images.

## Author contribution

Writing, editing, literature research: RS, HK, IG

Performed surgery: RS, AA

Performed diagnosis: AA, TW, MB, AL, DS

Editing: RS, AL, IG

All authors have read and approved the manuscript.

## Registration of research studies

Not applicable.

## Guarantor

Robert Sucher MD.

## Provenance and peer review

Not commissioned, externally peer-reviewed.

## Declaration of Competing Interest

The hyperspectral camera used for the measurements in this publication was developed by Diaspective Vision GmbH. H. Köhler is an employee of this company. The other authors declare no financial competing interests related to this work.
